# Current Evidence on Traditional Chinese Exercises for Quality of Life in Patients With Essential Hypertension: A Systematic Review and Meta-Analysis

**DOI:** 10.3389/fcvm.2020.627518

**Published:** 2021-01-20

**Authors:** Yang Song, Jialin Li, Bíró István, Rongrong Xuan, Sheng Wei, Guanghui Zhong, Yaodong Gu

**Affiliations:** ^1^Faculty of Sports Science, Ningbo University, Ningbo, China; ^2^Doctoral School of Safety and Security Sciences, Obuda University, Budapest, Hungary; ^3^Faculty of Engineering, University of Szeged, Szeged, Hungary; ^4^Medical School of Ningbo University, Ningbo University, Ningbo, China; ^5^The Affiliated Hospital of Medical School, Ningbo University, Ningbo, China; ^6^Ningbo Municipal Hospital of T.C.M., Ningbo, China

**Keywords:** essential hypertension, traditional Chinese exercises, quality of life, randomized controlled trial, meta-analysis

## Abstract

Essential hypertension is one of the most common chronic diseases seen in primary human health care that could lead to various health problems and reduce the quality of life (QOL). This study was performed to evaluate the effects of traditional Chinese exercises (TCE) on QOL in patients with essential hypertension. Three English databases and one Chinese database were searched for randomized controlled trials (RCTs) until August 2020. A total of 13 RCTs with 1,361 hypertensive patients met the inclusion criteria, 10 trials employed Tai Chi and 3 trials employed Qigong, including Dongeui Qinggong, Yijinjing, and Wuqinxi. Despite a large heterogeneity within studies, it is demonstrated that TCE may be an effective therapy to improve the QOL of hypertensive patients. More specific, compared with no intervention, the meta-analysis presented that Tai Chi significantly improved both the physical and mental component of the 36-item short-form health survey (SF-36) QOL scale, and it was found that the simplified 24-form Tai Chi with a frequency of 30–50 min per session at five to eight sessions per week was the most commonly used setting. The pooling also revealed that Tai Chi and Qigong had some significantly positive effects on QOL based on other scales. However, more rigorously designed RCTs are warranted for further verification. This systematic review and meta-analysis of RCTs demonstrated that Tai Chi may be an effective therapy to improve the QOL of patients with essential hypertension. All these findings provide helpful information for hypertensive patients and medical personnel.

## Introduction

Hypertension, also known as “the silent killer,” is a clinical syndrome that is generally characterized by the systemic elevated arterial pressure (1, 2). It is one of the most common chronic diseases seen in primary human health care and has been found to be the major independent risk factor that leads to cardio-cerebrovascular diseases (3, 4). There are two types of hypertension, primary hypertension and secondary hypertension, with the former being the most common one but the pathogenesis is still unclear. According to a research published on the *Journal of the American Medical Association* (*JAMA*), hypertension is responsible for at least 7.6 million deaths per year globally, equal to 13.5% of all deaths (5). In addition, it has been predicted that the total number of adults diagnosed with hypertension would increase by 60% to ~1.56 billion in 2025 (6), which puts a huge burden on global health care resources and the communities.

In early 2020, a novel coronavirus disease, i.e., corona virus disease 2019 (COVID-19), has swept across the globe, caused thousands of deaths and millions of economic loss (7, 8). The situation now has not been controlled, where the number of infected people is still increasing greatly every day. According to the latest official report published by the World Health Organization (WHO), there have been 24,299,923 confirmed cases of COVID-19, including 827,730 deaths globally by 4:33 pm CEST, 28 August 2020 (9). In order to restrain the human-to-human spread of COVID-19, most of the governments have suggested the citizens to stay at home (10). However, although home isolation can be effective in infection control, it may also bring some potential health threats. Emerging studies indicated that home isolation during this period has led to a substantial decrease in physical activity levels and a profound increase in sedentary behavior worldwide, which may rapidly deteriorate cardiovascular health, increase the incidence of hypertension, and further exacerbate the global burden (11, 12).

Despite the continuous exploration of hypertension treatment during the past decades, the main therapy for essential hypertension is still pharmacological management—taking antihypertensive drugs regularly (1, 13). However, the effectiveness and patient compliance are always limited because of the high cost and several adverse effects, such as long-term medication and hypokalemia (1, 14). Therefore, alternative therapies with greater efficiency and no side effect are much warranted.

In recent years, traditional Chinese exercises (TCE) have gained popularity worldwide because of their benefit on improving both physical and mental health in patients with chronic diseases (15). TCE are comprehensive mind–body exercises that incorporate appropriate body movement, breathing patterns, and meditation (16). For example, Tai Chi, which now basically consists of 24 moves, emphasizes the unity of relaxation and dynamic balance. It can help to enhance the muscle strength, joint stability, and relieve the pressure. Wuqinxi, which is a set of TCE that imitates the movement of tiger, deer, bear, ape, and bird, focuses on the combination of dynamic and static or inside and outside, and could regulate the function of the internal organs. An increasing number of studies have investigated the effects of TCE on patients with essential hypertension and most found that TCE are effective in reducing diastolic and systolic blood pressure although there are some differences in experimental design (17, 18). Also, there are several systematic reviews and meta-analysis that have further summarized and confirmed these advantages (3, 19, 20). Nevertheless, the effects of TCE on quality of life (QOL) in patients with essential hypertension were not covered in previous studies. QOL is a broad-ranging concept that emphasizes the measurement and scoring of a person's multiple aspects, including perceived physical health, mental state, beliefs, social relations, etc., which can be used for the comprehensive assessment of patients with essential hypertension (21). Thus, the purpose of this study was to systematically review and evaluate the effects of TCE on QOL in patients with essential hypertension.

## Methods

### Protocol Registration

This systematic review and meta-analysis was conducted based on the Preferred Reporting Items for Systematic Reviews and Meta Analyses (PRISMA) guidelines, and the PRISMA registration number is CRD42020218981.

### Inclusion Criteria

To be included into this systematic review, previous studies must meet the following eligibility criteria in accordance with PICOS.

#### Participants

Participants who are over 18 years old and have been diagnosed with essential hypertension based on the past or current definitions of hypertension were included, while there is no limit on sex and ethnicity. However, participants with serious organic disease and its complications were not included.

#### Interventions

TCE were limited to a few common ones, such as Tai Chi, Qigong, Baduanjin, Wuqinxi, Yijinjing, and Liuzijue. Both single TCE interventions and TCE interventions with health education were considered.

#### Comparison

There was no restriction regarding the control group (e.g., health education, behavioral regulation, and routine lifestyle were all treated as a homogeneously no-intervention group) but studies comparing the TCE interventions with other exercise modalities, such as aerobic and resistance exercise, were excluded.

#### Outcome

The primary outcome collected and analyzed from the included studies was QOL based on different types of scales.

#### Study Design

Only randomized controlled trials (RCTs) investigating the effects of TCE on QOL in patients with essential hypertension were considered; cross-sectional studies, observational studies, conference papers, etc., were not included. Because TCE are primarily originated and developed in China, both Chinese and English papers published on peer-reviewed journals were covered.

### Search Strategy and Data Sources

A systematic literature search was performed in the following four online electronic databases to identify all the relevant studies from their inception until 10 August 2020: Google Scholar (all years), Web of Science (1960-present), ScienceDirect (all years), and China National Knowledge Infrastructure (all years, available at https://www.cnki.net/). The following search terms were used in English databases: (“essential hypertension” AND “quality of life”) AND (“Tai Chi” OR “Qigong” OR “Baduanjin” OR “Wuqinxi” OR “Yijinjing” OR “Liuzijue” OR “traditional Chinese exercise”), while their Chinese counterparts were used in the Chinese database. The search strategy was slightly modified in order to suit each database.

In order to ensure a rigorous and thorough literature search, two authors independently screened and assessed all the retrieved articles from the databases based on the included criteria. Any disagreements regarding the inclusion would be discussed and resolved with the third author. Moreover, all reference lists from the included studies and the retrieved reviews were double-checked using the citation snowballing method to ensure that all the potential relative articles were located (22, 23).

### Data Extraction and Management

Two authors independently screened, extracted, and summarized the following relevant data from all the original articles: (1) the basic characteristics of included studies (authors, country where the trial was performed, and publication year); (2) the basic characteristics of participants (number, age, gender, blood pressure, and diagnosis of hypertension); (3) study intervention (the types of exercise, frequency, and duration); and (4) outcome parameters and primary results. Any differences regarding data extraction would be discussed and resolved with the third author. Mendeley Reference Management Software (Mendeley Ltd., Netherlands) was used to organize papers and generate citations.

### Risk of Bias Assessment

Two authors independently assessed the risk of bias of all the included studies using the Cochrane Risk of Bias Assessment Tool, which includes the following seven domains: random sequence generation, allocation concealment, blinding of participants and personnel, blinding of outcome assessment, incomplete outcome data, selective reporting, and other biases (24). There are three grades for each domain: low risk of bias, unclear risk of bias, and high risk of bias. Any disagreements regarding the risk of bias assessment would be discussed and resolved with the third author.

### Statistical Analysis

The Review Manager software (RevMan 5.4, The Cochrane Collaboration, 2020) was applied to perform the meta-analysis. Because the outcomes from the included studies were all continuous, they were presented as the mean difference (MD, applicable if data in the same unit) or standardized mean difference (SMD, applicable otherwise) and 95% confidence intervals (95% CI). The Chi-square (χ^2^) test and *I*-square (*I*^2^) statistic were used to assess heterogeneity among studies, and it was considered as significant when *I*^2^ > 50%. The outcome data from each included studies were combined through a meta-analysis by using the random effects model. Firstly, the overall effect of TCE on QOL was determined using the difference before and after intervention, and then subgroups were further created based on the types and duration of TCE interventions. In addition, the publication bias was evaluated using the funnel plot asymmetry if 10 trials (at least) were covered in a meta-analysis. Results were considered statistically significant when *p* < 0.05.

## Results

### Literature Search Results

**Figure 1:**
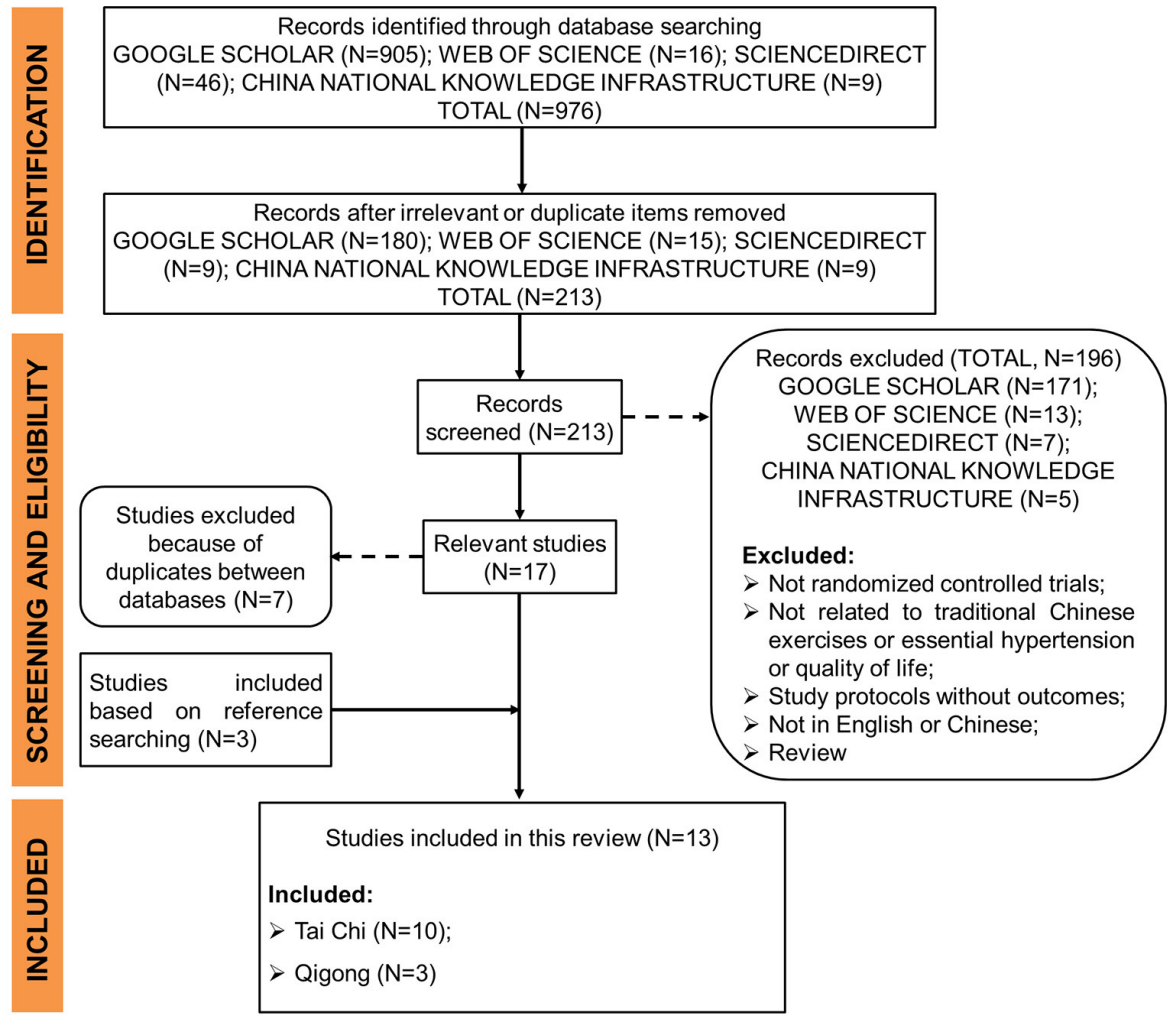
Flow diagram of literature search and selection process.

The literature search and selection process are presented in Figure 1. A total of 976 studies were identified through database searching. After removing 763 irrelevant or duplicate records based on titles, the remaining 213 studies were further evaluated according to the eligibility criteria. Then, 171 papers from Google Scholar, 13 papers from Web of Science, 7 from ScienceDirect, and 5 from China National Knowledge Infrastructure were excluded for the following several reasons (e.g., the experimental design is not randomized, studies not related to TCE or essential hypertension or QOL, study protocols without outcomes, studies not written in English or Chinese, and review studies). Seven studies were excluded because of duplicates between databases, while three studies were further eligible for inclusion after double-checking the references of all the included studies and the retrieved systematic reviews. In total, 13 studies were finally included in this review.

### Risk of Bias

**Figure 2:**
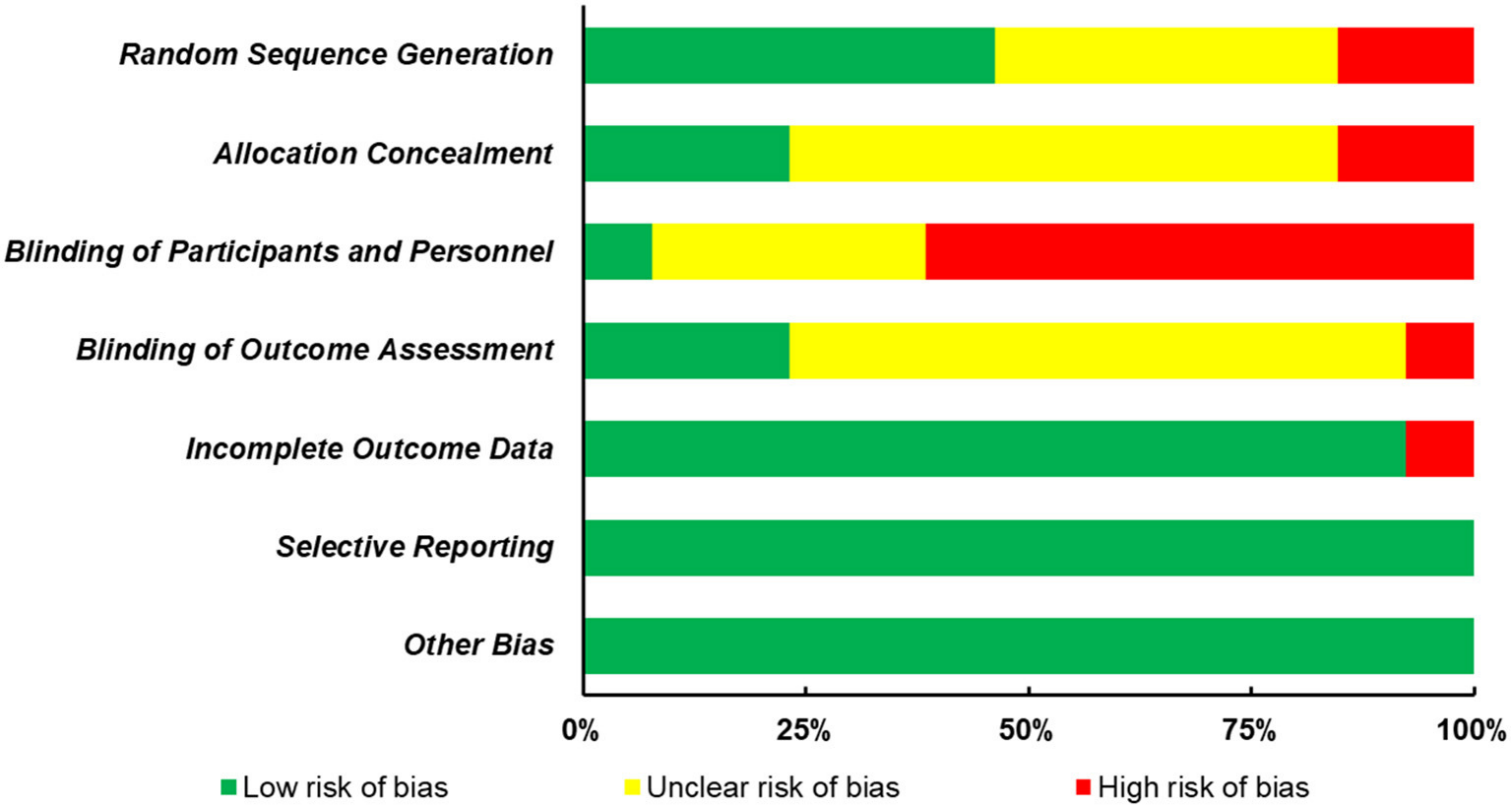
Risk of bias graph.

The quality of all selected studies was assessed in terms of risk of bias, and the results were presented in Figure 2 and Table 1. Less than half of the included studies (*n* = 6) clearly described the process of random sequence generation. Two studies that allocated participants based on the order of hospitalization were judged to have high risk of bias, while the remaining studies (*n* = 5) were judged as unclear risk of bias as none of them gave the detailed procedure. Regarding the allocation concealment, three studies conceal the allocation from both researchers and participants using opaque envelope, etc., eight studies did not clearly describe the allocation concealment, while two studies did not conceal the allocation from both researchers and participants. In addition, since there is less trial reported to blind their participants, personnel, and outcome assessors or they did not blind them, these two domains were also the major sources that increase the risk of bias. However, a low risk of bias was reported in most studies for the incomplete outcome data (*n* = 12) and selective reporting (*n* = 13), and there were no other biases described in these studies.

**Table 1:** Risk of bias summary.

**Trials**	**Random sequence generation**	**Allocation concealment**	**Blinding of participants and personnel**	**Blinding of outcome assessment**	**Incomplete outcome data**	**Selective reporting**	**Other bias**
Gou et al. (25)	High	High	High	Unclear	Low	Low	Low
Han et al. (26)	Unclear	Unclear	Unclear	Unclear	Low	Low	Low
Hong et al. (27)	Unclear	Unclear	High	Unclear	Low	Low	Low
Li (28)	High	High	High	Unclear	Low	Low	Low
Liu et al. (29)	Low	Unclear	High	Unclear	Low	Low	Low
Ma et al. (30)	Low	Low	High	Low	High	Low	Low
Park et al. (31)	Low	Low	High	Low	Low	Low	Low
Shou et al. (32)	Unclear	Unclear	Unclear	Unclear	Low	Low	Low
Sun et al. (33)	Unclear	Low	Low	Low	Low	Low	Low
Xiao et al. (34)	Low	Unclear	High	Unclear	Low	Low	Low
Xu et al. (35)	Low	Unclear	Unclear	Unclear	Low	Low	Low
Zhang (36)	Low	Unclear	Unclear	Unclear	Low	Low	Low
Zheng et al. (37)	Unclear	Unclear	High	High	Low	Low	Low

### Study Characteristics

The basic characteristics of all the included studies are shown in Table 2. In the 13 included RCTs published from 2010 to 2019, most of them were conducted in People's Republic of China (*n* = 11, 84.62%), and the remaining two studies were conducted in Republic of Korea and Australia, respectively (*n* = 1, 7.69%). Four studies were published in English, while the other nine were published in Chinese. The 13 studies involved a total of 1,361 patients aged 30–80 years old (681 in the intervention group and 680 in the control group), and all studies (*n* = 13, 100%) included both male and female participants. The severity of essential hypertension varied between studies, with diastolic blood pressure (DBP) ranging from 80 to 110 mmHg and systolic blood pressure (SBP) ranging from 130 to 180 mmHg. Three diagnostic criteria for essential hypertension were found used in these included studies. Regarding the types of TCE intervention, Tai Chi was applied in 10 studies, and 24-form Yang style Tai Chi was the most common one. Qigong was applied in three studies, including Dongeui Qinggong, Yijinjing, and Wuqinxi, and they were considered as a homogeneous TCE intervention as the theory and principles are similar among them. The treatment duration ranged from 2 months to 5 years, and the common session lasted about 30 to 60 min, with 5 to 8 sessions per week. Seven scales were used to assess the QOL, with the 36-item Short-Form Health Survey (SF-36) being the most common one. Results were further examined in the following three parts: (1) overall effects of TCE on QOL, (2) effects of Tai Chi on QOL, and (3) effects of Qigong on QOL.

**Table 2:** The basic characteristics of included studies.

**Study**	**Country/Region**	**Sample size (finished, *N*)**	**Gender**	**Age (years)**	**Blood pressure**	**Diagnosis of hypertension**	**Intervention**	**Control**	**Frequency and duration**	**Outcome parameters**
			**M/F**		**SBP/DBP (mmHg)**					
Gou et al. (25)	China	*N*: 108 TCE: 54 CON: 54	TCE: 30/24 CON: 31/23	TCE: 75.38 ± 5.69 CON: 74.29 ± 4.58	NA	NA	Tai Chi	Conventional therapy	6 months (patient customization)	Quality of life (GQOL-74)
Han et al. (26)	China	*N*: 58 TCE: 30 CON: 28	38/22	62.12 ± 10.51	163.1 ± 8.2/ 100.2 ± 9.1	WHO/ISH	Simplified 24-form Tai Chi	Conventional therapy	1–2 sessions per day (45–60 min per session) for 5 years	Quality of life (SF-36)
Hong and Wang (27)	China	*N*: 50 TCE: 25 CON: 25	NA	NA	TCE: 134.08 ± 8.87/88.32 ± 5.99 CON: 131.88 ± 6.61/89.56 ± 4.88	NA	Traditional Shaolin Yijinjing	Routine lifestyle	7 sessions per week (1.5 h per session) for 12 weeks	Quality of life (WHOQOL-BREF)
Li (28)	China	*N*: 122 TCE: 61 CON: 61	TCE: 30/31 CON: 33/28	TCE: 51.08 ± 8.77 CON: 50.51 ± 8.68	TCE: 161.58 ± 18.24/110.51 ± 12.36 CON: 158.61 ± 22.13/107.94 ± 12.78	NA	Simplified 24-form Tai Chi	Conventional therapy	5–8 sessions per week (50–60 min per session) for 12 weeks	Quality of life (SF-36)
Liu et al. (29)	China	*N*: 70 TCE: 35 CON: 35	TCE: 18/17 CON: 19/16	TCE: 62.4 ± 2.4 CON: 63.1 ± 2.1	TCE: 157.96 ± 15.24/97.24 ± 6.84 CON: 158.45 ± 15.73/97.85 ± 6.58	Guidelines for Prevention and Treatment of Hypertension in China (2010 revised version)	Simplified 24-form Tai Chi	Conventional therapy	1 session (40–60 min) per day for 1 year	Quality of life (SF-36)
Ma et al. (30)	China	*N*: 113 TCE: 55 CON: 58	TCE: 54/25 CON: 55/24	TCE: 70.24 ± 10.25 CON: 69.71 ± 10.84	TCE: 149.06 ± 19.51/90.74 ± 8.24 CON: 150.19 ± 18.30/89.16 ± 9.37	NA	Simplified 24-form Tai Chi	Usual care	3–5 sessions per week (60 min per session) for 24 weeks	Health-related quality of life (SF-36)
Park et al. (31)	Republic of Korea	*N*: 52 TCE: 25 CON: 27	TCE: 13/12 CON: 22/5	TCE: 54.52 ± 6.96 CON: 52.93 ± 8.45	TCE: 134.45 ± 10.41/85.23 ± 6.43 CON: 130.47 ± 13.93/ 85.41 ± 7.83	NA	Dongeui Qigong	Routine lifestyles	5–7 sessions per week (50 min per session) for 12 weeks	Quality of life (MYMOP)
Shou et al. (32)	China	*N*: 198 TCE: 98 CON: 100	TCE: 48/50 CON: 55/45	TCE: M: 52 ± 6.46; F: 51 ± 7.09 CON: M: 52 ± 8.98; F: 51 ± 7.54	TCE: 139.42 ± 9.47/ 83.20 ± 9.45 CON: 142.90 ± 7.91/ 82.40 ± 7.82	Guidelines for Prevention and Treatment of Hypertension in China (2010 revised version)	Simplified 24-form Tai Chi	General daily lifestyle intervention	1–2 sessions per day (40–90min per session) for 3 months	Quality of life (SF-36)
Sun et al. (33)	Australia	*N*: 266 TCE: 136 CON: 130	TCE: 19/117 CON: 29/101	45–80	TCE: 130.71 ± 16.65/82.21 ± 7.94 CON: 130.46 ± 15.97/81.92 ± 8.25	World Diabetes Association standard 2002	Tai Chi	Non-exercise-related activities	3-h group exercise and 2-h home exercise per week for 12 months	Health-related quality of life (SF-36)
Xiao et al. (34)	China	*N*: 84 TCE: 42 CON: 42	TCE: 24/18 CON: 22/20	TCE: 60.2 ± 4.6 CON: 60.5 ± 4.9	TCE: 151.4 ± 10.3/90.8 ± 10.5 CON: 151.8 ± 10.2/90.4 ± 10.2	Guidelines for Prevention and Treatment of Hypertension in China (2010 revised version)	Eight-style Tai Chi	Conventional therapy	5 sessions per week (60 min per session) for 3 months	Quality of life (SF-36)
Xu et al. (35)	China	*N*: 60 TCE: 30 CON: 30	TCE: 17/13 CON: 18/12	TCE: 38.07 ± 8.09 CON: 37.63 ± 9.09	TCE: 158.4 ± 8.8/97.5 ± 4.2 CON: 159.1 ± 7.5/96.7 ± 5.0	WHO/ISH (4th revised version)	Simplified 24-form Tai Chi	Conventional therapy	2 sessions per day (10 min per session) for 8 weeks	Quality of life (SF-36)
Zhang. (36)	China	*N*: 82 TCE: 41 CON: 41	TCE: 26/15 CON: 25/16	TCE: 55.2 ± 5.3 CON: 55.3 ± 5.1	NA	NA	Wuqinxi	Conventional therapy	1 session per day (1.5 h per session) for 6 months	Quality of life (PRO)
Zheng et al. (37)	China	*N*: 98 TCE: 49 CON: 49	TCE: 20/29 CON: 19/30	TCE: 54.71 ± 5.43 CON: 55.77 ± 6.24	TCE: 157.96 ± 15.24/97.24 ± 6.84 CON: 158.45 ± 15.73/97.85 ± 6.58	Guidelines for Prevention and Treatment of Hypertension in China (2010 revised version)	Simplified 24-form Tai Chi	Conventional therapy	4–8 sessions per week (40–60 min per session) for 3 months	Quality of life (Leishmania hypertension scale)

*N, number; M, male; F, female; SBP, systolic blood pressure; DBP, diastolic blood pressure; TCE, traditional Chinese exercises; CON, control interventions; NA, not available; WHO/ISH, World Health Organization/International Society of Hypertension; GQOL-74, Generic Quality of Life Inventory-74; SF-36, 36-Item Short-Form Health Survey; WHOQOL-BREF, World Health Organization Quality of Life; MYMOP, Measure Yourself Medical Outcome Profile; PRO, Patient-Reported Outcomes*.

### Overall Effects of TCE on QOL

**Figure 3:**
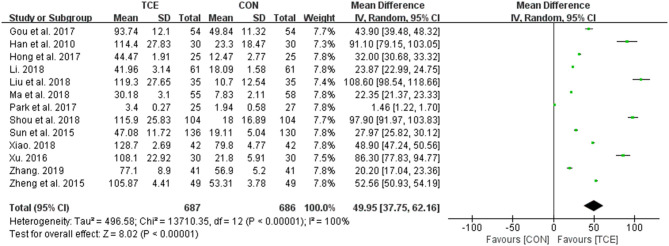
Meta-analysis of the overall effects of TCE on QOL vs. the control intervention. TCE, traditional Chinese exercises; CON, control; SD, standard deviations; CI, confidence intervals; IV, inverse variance.

In the comparison of the overall effects of TCE and control interventions on QOL, 13 studies were covered for the meta-analysis. After pooling the findings of all these studies, it was indicated that TCE did help to improve the QOL among people with essential hypertension irrespective of all the other variables (types of TCE and QOL scales, and the number/duration of sessions) although the heterogeneity is extremely high (MD = 49.95; 95% CI: 37.75–62.16; *p* < 0.00001; *I*^2^ = 100%). The forest plot of the pooled effects is shown in Figure 3.

### Effects of Tai Chi on QOL

A total of 10 studies investigating the effects of Tai Chi on QOL in patients with essential hypertension were included in this category. SF-36 scale was used in 8 studies (26, 28–30, 32–35) while the Leishmania Hypertension scale and the Generic Quality of Life Inventory (GQOL) scale were used in the remaining two studies (25, 37) to assess QOL, respectively.

**Figure 4:**
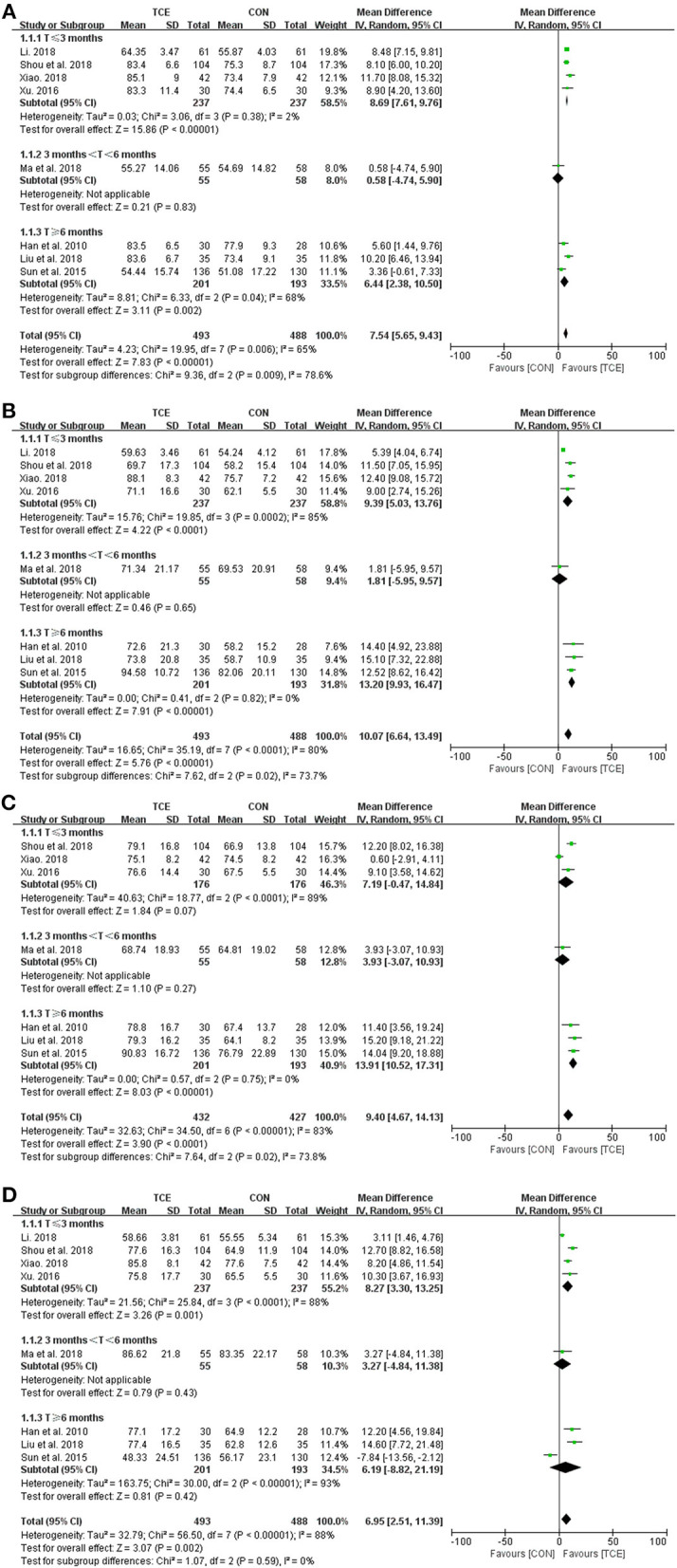
Meta-analysis of SF-36 QOL (physical component) in the TCE group (Tai Chi) vs. the control group. **(A)** Physical function; **(B)** role physical; **(C)** bodily pain; and **(D)** general health. TCE, traditional Chinese exercises; CON, control; SD, standard deviations; CI, confidence intervals; IV, inverse variance.

**Figure 5:**
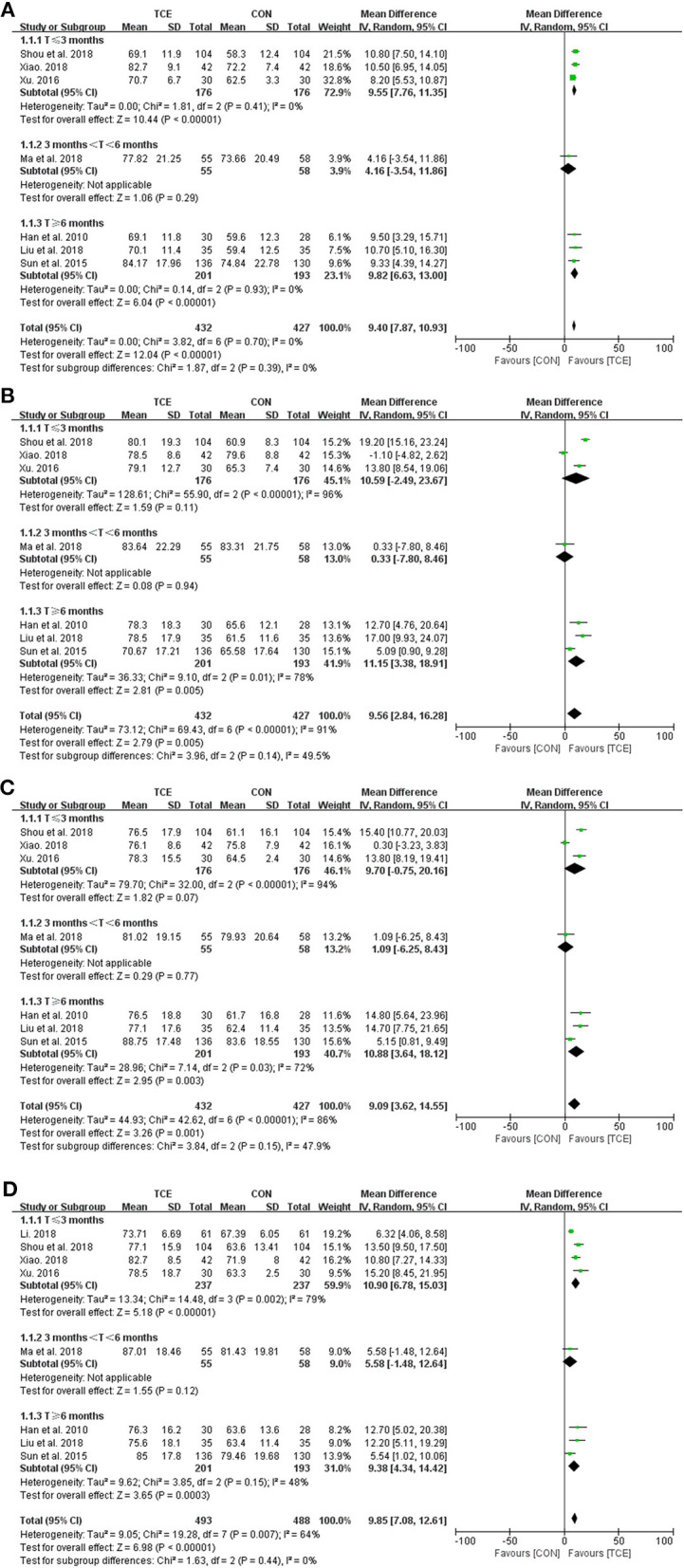
Meta-analysis of SF-36 QOL (mental component) in the TCE group (Tai Chi) vs. the control group. **(A)** Vitality; **(B)** social function; **(C)** role emotional; and **(D)** mental health. TCE, traditional Chinese exercises; CON, control; SD, standard deviations; CI, confidence intervals; IV, inverse variance.

There are two components in the SF-36 scale, with the physical component scale including physical function, role physical, bodily pain, and general health domains and the mental component scale including vitality, social function, role emotional, and mental health domains. Using the SF-36 scale in eight studies, which involve 981 participants, the meta-analysis found that Tai Chi can significantly improve every domain of the physical component scale [physical function (MD = 7.54; 95% CI: 5.65–9.43; *p* < 0.00001; *I*^2^ = 65%), role physical (MD = 10.07; 95% CI: 6.64 to 13.49; *p* < 0.00001; *I*^2^ = 80%), bodily pain (MD = 9.40; 95% CI: 4.67–14.13; *p* < 0.0001; *I*^2^ = 83%), and general health (MD = 6.95; 95% CI: 2.51–11.39; *p* = 0.002; *I*^2^ = 88%)] and the mental component scale [vitality (MD = 9.40; 95% CI: 7.87–10.93; *p* < 0.00001; *I*^2^ = 0%), social function (MD = 9.56; 95% CI: 2.84–16.28; *p* = 0.005; *I*^2^ = 91%), role emotional (MD = 9.09; 95% CI: 3.62–14.55; *p* = 0.001; *I*^2^ = 86%), and mental health domains (MD = 9.85; 95% CI: 7.08–12.61; *p* < 0.00001; *I*^2^ = 64%)] when compared with the control group (Figures 4, 5). A subgroup meta-analysis was conducted between Tai Chi and the control group to examine if the heterogeneity could be partially explained by the different duration of the interventions; thus, the duration below 3 months was considered as short term, between 3 and 6 months was considered as medium term, and above 6 months was considered as long term. However, the heterogeneity was not resolved since there are still significant heterogeneities in these trials, with *I*^2^ ranging from 0 to 96% (Figures 4, 5).

One study, involving 98 participants, examined the effects of Tai Chi on QOL based on the Leishmania Hypertension scale (37). Using the random effects model, it was found that all the 11 domains were significantly lower in participants performing Tai Chi than their counterparts in the control group [physical symptom (MD = −10.77; 95% CI: −13.45 to −8.09; *p* < 0.00001), somatization symptom (MD = −10.80; 95% CI: −11.79 to −9.81; *p* < 0.00001), sexual function (MD = −4.40; 95% CI: −4.99 to −3.81; *p* < 0.00001), sleep quality (MD = −1.43; 95% CI: −2.23 to −0.63; *p* = 0.0005), angry/vitality (MD = −4.85; 95% CI: −5.33 to −4.37; *p* < 0.00001), anxiety (MD = −4.14; 95% CI: −4.81 to −3.47; *p* < 0.00001), repression (MD = −2.98; 95% CI: −3.63 to −2.33; *p* < 0.00001), obsession (MD = −3.23; 95% CI: −3.82 to −2.64; *p* < 0.00001), interpersonal relationships (MD = −3.88; 95% CI: −4.63 to −3.13; *p* < 0.00001), working order (MD = −5.71; 95% CI: −6.26 to −5.16; *p* < 0.00001), and hostility (MD = −4.15; 95% CI: −5.12 to −3.18; *p* < 0.00001)]. Similarly, the remaining one study investigating the effects of Tai Chi on QOL based on GQOL also reached positive results (25). Tai Chi significantly improved all the four domains vs. no intervention [physical function (MD = 10.45; 95% CI: 8.17–12.73; *p* < 0.00001), mention function (MD = 11.20; 95% CI: 9.02–13.38; *p* < 0.00001), social function (MD = 10.47; 95% CI: 8.32–12.62; *p* < 0.00001), and material life state (MD = 11.78; 95% CI: 9.55–14.01; *p* < 0.00001)] (Table 3). Nevertheless, the quality of these two studies was found to be relatively poor, which may weaken the reliability of the results.

**Table 3:** Summary of meta-analysis based on other QOL scales.

**Parameters**	**Tai Chi vs. no intervention**					**Parameters**	**Qigong vs. no intervention**				
	**Trial (*n*)**	**Participants (*n*)**	**Mean difference (95% CI)**	**Heterogeneity**	***p*-value**		**Trial (*n*)**	**Participants (*n*)**	**Mean difference (95% CI)**	**Heterogeneity**	***p*-value**
**Leishmania hypertension scale**						**MYMOP**					
Physical symptom	1	98	−10.77 [−13.45, −8.09]	NA	<0.00001	Total	1	52	−0.44 [−1.04, 0.16]	NA	0.15
Somatization symptom	1	98	−10.80 [−11.79, −9.81]	NA	<0.00001	Symptoms	1	52	−0.50 [−1.26, 0.26]	NA	0.20
Sexual function	1	98	−4.40 [−4.99, −3.81]	NA	<0.00001	Activity	1	52	1.83 [1.17, 2.49]	NA	<0.00001
Sleep quality	1	98	−1.43 [−2.23, −0.63]	NA	0.0005	Wellbeing	1	52	−1.00 [−1.62. −0.38]	NA	0.001
Angry/vitality	1	98	−4.85 [−5.33, −4.37]	NA	<0.00001	**WHOQOL-BREF**					
Anxiety	1	98	−4.14 [−4.81, −3.47]	NA	<0.00001	Physical health	1	50	15.43 [12.21, 18.65]	NA	<0.00001
Repression	1	98	−2.98 [−3.63, −2.33]	NA	<0.00001	Psychological health	1	50	12.34 [9.31, 15.37]	NA	<0.00001
Obsession	1	98	−3.23 [−3.82, −2.64]	NA	<0.00001	Social relationships	1	50	2.33 [−3.81, 8.47]	NA	0.46
Interpersonal relationships	1	98	−3.88 [−4.63, −3.13]	NA	<0.00001	Environment	1	50	−2.12 [−5.73, 1.49]	NA	0.25
Working order	1	98	−5.71 [−6.26, −5.16]	NA	<0.00001	**PRO**					
Hostility	1	98	−4.15 [−5.12, −3.18]	NA	<0.00001	Total	1	82	−18.80 [−24.56, −13.04]	NA	<0.00001
**GQOL-74**											
Physical function	1	108	10.45 [8.17, 12.73]	NA	<0.00001						
Mention function	1	108	11.20 [9.02, 13.38]	NA	<0.00001						
Social function	1	108	10.47 [8.32, 12.62]	NA	<0.00001						
Material life state	1	108	11.78 [9.55, 14.01]	NA	<0.00001						

*N, number; CI, confidence intervals; NA, not applicable; GQOL-74, Generic Quality of Life Inventory-74; WHOQOL-BREF, World Health Organization Quality of Life; MYMOP, Measure Yourself Medical Outcome Profile; PRO, Patient-Reported Outcomes*.

### Effects of Qigong on QOL

Three more studies were included in this review, which evaluated the effects of Qigong on QOL based on different scales (27, 31, 36). As it is shown in Table 3, using the World Health Organization Quality of Life (WHOQOL-BREF) scale, those who perform Qigong showed significant differences in two domains when compared to no-intervention participants [physical health (MD = 15.43; 95% CI: 12.21–18.65; *p* < 0.00001) and psychological health (MD = 12.34; 95% CI: 9.31–15.37; *p* < 0.00001)]. This suggested that Qigong improves both the physical and psychological health. Using the Measure Yourself Medical Outcome Profile (MYMOP) scale, the meta-analysis indicated that Dongeui Qigong resulted in a significant improvement in the activity (MD = 1.83; 95% CI: 1.17–2.49; *p* < 0.00001) but a decline in the well-being (MD = −1.00; 95% CI: −1.62 to −0.38; *p* = 0.001) subscale. In addition, when the Patient-Reported Outcomes (PRO) scale is applied, the meta-analysis showed that Wuqinxi Qigong resulted in a significantly lower total score (WMD = −18.80; 95% CI: −24.56 to −13.04; *p* < 0.001) when compared to the control group, which indicated that Wuqinxi Qigong improved the QOL overall. In general, although the study quality was moderate, more trials using these above scales to evaluate the effects of TCE on QOL are warranted.

## Discussion

TCE are comprehensive mind–body exercises that have been widely applied to treat patients with hypertension. Previous systematic review and meta-analysis mainly focused on some specific types of TCE and investigated their effects on blood pressure. The current study systematically reviewed the previous literature and provided an objective evaluation on the effects of TCE on QOL in hypertensive patients, with the aim of finding out the appropriate TCE that can contribute to more benefits.

Following the eligibility criteria, 13 RCTs comprising a total of 1,361 patients with mild-to-severe essential hypertension were included in this review. Although there is considerable heterogeneity within the included studies, the overall effect was positive with a statistical significance, indicating that TCE had an advantage over control intervention on improving QOL of hypertensive patients. Then, a further detailed subgroup analysis based on different QOL scales and duration of TCE interventions revealed different outcomes regarding the use of TCE. Compared with conventional therapy/usual care, Tai Chi had significantly greater effects on both physical and mental components of SF-36 scale. To sum up, the benefits of Tai Chi on SF-36 QOL were consistent among studies, and it was found that the simplified 24-form Tai Chi with a frequency of 30–50 min per session at five to eight sessions per week was the most commonly used setting. The potential mechanisms by which TCE adds greater help for improving QOL of hypertensive patients have been speculated in previous studies. For example, Tai Chi is a type of TCE that focuses on three aspects, dynamic balance of physical movement, which enhances muscle strength and joint stability; dynamic balance of breathing flow, which improves the depth and regularity of breathing; and psychological regulation, which reduces mental stress (1, 16, 38). Thus, the enhanced QOL after Tai Chi may be associated with the overall improvements of the above three parts.

In addition to the effects of Tai Chi intervention on SF-36 QOL, there are also two studies that investigated the effects of Tai Chi on QOL based on other scales (25, 37); three studies that investigated the effects of Dongeui Qigong, Yijinjing, and Wuqinxi on QOL based on several scales (27, 31, 36); and the meta-analysis indicated that all of these TCE interventions could improve the QOL to a certain degree. However, due to the limited number of studies in each group and the relatively poor study quality, the reanalyzed results may not be accurate and should be treated with caution. A greater number of studies are much warranted for further verification.

Some potential limitations of this study and the design flaws of these included studies may weaken the reliability of the results. For example, only RCTs written in English or Chinese were included in this review. Small sample size, relatively high risk of bias, heterogeneity, and relatively weak methodology of these selected trials are other problems. In addition, there are only a small number of studies in some subgroups, which potentially results in an overestimation of effect sizes when conducting the meta-analysis. Lastly, studies that found neutral or negative results are not often published, which may also weaken the accuracy of meta-analysis.

## Conclusions

In conclusion, the results of this systematic review and meta-analysis demonstrated that TCE, especially Tai Chi, could be an effective therapy to improve the QOL of patients with essential hypertension compared to conventional therapy. However, more rigorously designed RCTs are warranted for further verification, and the effects of other types of TCE interventions on QOL remain to be further clarified.

## Data Availability Statement

The original contributions generated for the study are included in the article/Supplementary Material, further inquiries can be directed to the corresponding author/s.

## Author Contributions

YS, JL, and SW conceived the presented idea, developed the framework, and wrote the manuscript. BI, RX, GZ, and YG provided critical feedback and contributed to the final version. All authors were involved in the final direction of the paper and contributed to the final version of the manuscript. All authors have read and agreed to the published version of the manuscript.

## Conflict of Interest

The authors declare that the research was conducted in the absence of any commercial or financial relationships that could be construed as a potential conflict of interest.
